# Inflamação Coronária Avaliada pela Atenuação de Gordura Pericoronária na Tomografia Computadorizada e Elevação de Citocinas em Usuários Jovens de Esteroides Anabólicos Androgênicos

**DOI:** 10.36660/abc.20220822

**Published:** 2023-11-13

**Authors:** Francis Ribeiro de Souza, Carlos E. Rochitte, Douglas Carli Silva, Barbara Sampaio, Marisa Passarelli, Marcelo R. dos Santos, Guilherme W. Fonseca, Antonio Carlos Battaglia, Kelly Correa, Renata Margarida do Val, Maurício Yonamine, Rosa Maria R. Pereira, Carlos Eduardo Negrão, Roberto Kalil-Filho, Maria Janieire de Nazaré Nunes Alves

**Affiliations:** 1 Hospital das Clínicas Faculdade de Medicina Universidade de São Paulo São Paulo SP Brasil Instituto do Coração do Hospital das Clínicas da Faculdade de Medicina da Universidade de São Paulo , São Paulo , SP – Brasil; 2 Faculdade de Medicina Universidade de São Paulo São Paulo SP Brasil Faculdade de Medicina da Universidade de São Paulo (FMUSP), São Paulo , SP – Brasil; 3 Instituto de Medicina Tropical de São Paulo São Paulo SP Brasil Instituto de Medicina Tropical de São Paulo , São Paulo , SP – Brasil; 4 Universidade de São Paulo Faculdade de Medicina Laboratório de Lípides São Paulo SP Brasil Universidade de São Paulo – Faculdade de Medicina – Laboratório de Lípides , São Paulo , SP – Brasil; 5 Universidade Nove de Julho São Paulo SP Brasil Universidade Nove de Julho , São Paulo , SP – Brasil; 6 Hospital Israelita Albert Einstein São Paulo SP Brasil Hospital Israelita Albert Einstein , São Paulo , SP – Brasil; 7 Universidade de São Paulo Faculdade de Ciências Farmacêuticas São Paulo SP Brasil Universidade de São Paulo – Faculdade de Ciências Farmacêuticas , São Paulo , SP – Brasil; 8 Hospital Sírio Libanês São Paulo SP Brasil Hospital Sírio Libanês , São Paulo , SP – Brasil

**Keywords:** Doença da Artéria Coronariana, Tomografia Computadorizada por Raios X, Esteroide Anabólico Androgênico

## Abstract

**Fundamento:**

O uso abusivo de esteroides anabólicos androgênicos (EAA) tem sido associado à doença arterial coronariana (DAC). A atenuação de gordura pericoronária (AGp) é um marcador de inflamação coronária, a qual exerce um papel chave no processo aterosclerótico.

**Objetivo:**

Avaliar AGp e perfil inflamatório em usuários de EAA.

**Método:**

Vinte indivíduos que realizavam treinamento de força, usuários de EAA (UEAA), 20 não usuários de EAA (NUEAA), e 10 indivíduos sedentários controle (SC) foram avaliados. Inflamação coronária foi avaliada por atenuação de gordura pericoronária média (AGPm) artéria coronária direita (ACD), artéria descendente anterior esquerda (ADA) e artéria circunflexa (ACX). Interleucina (IL)-1 (IL-1), IL-6, IL-10, e TNF-alfa foram avaliados por densidade ótica (DO) em um espectrofotômetro com um filtro de 450 nm. Um p<0,05 indicou significância estatística.

**Resultados:**

Os UEAA apresentaram maior AGPm na ACD [-65,87 (70,51-60,70) vs. -78,07 (83,66-72,87) vs.-78,46 (85,41-71,99] unidades Hounsfield (HU), respectivamente, p<0,001) e AGPm na ADA [-71,47 (76,40-66,610 vs. -79,32 (84,37-74,59) vs. -82,52 (88,44-75,81) HU, respectivamente, p=0,006) em comparação aos NUEAA e CS. A AGPm na ACX não foi diferente entre os grupos UEAA, NUEAA e CS [-72,41 (77,17-70,37) vs. -80,13 (86,22-72,23) vs. -78,29 (80,63-72,29) HU, respectivamente, p=0,163). Em comparação aos NUEAA e aos CS, o grupo UEAA apresentaram maiores níveis de IL-1 [0,975 (0,847-1,250) vs. 0,437 (0,311-0,565) vs. 0,530 (0,402-0,780) DO, respectivamente, p=0,002), IL-6 [1,195 (0,947-1,405) vs. 0,427 (0,377-0,577) vs. 0,605 (0,332-0,950) DO, p=0,005) e IL-10 [1,145 (0,920-1,292) vs. 0,477 (0,382-0,591) vs. 0,340 (0,316-0,560) DO, p<0,001]. TNF-α não foi diferente entre os grupos UEAA, NUEAA e CS [0,520 (0,250-0,610) vs. 0,377 (0.261-0,548) vs. 0,350 (0,182-430)].

**Conclusão:**

Em comparação aos NUEAA e controles, os UEAA apresentam maior AGPm e maior perfil de citocinas inflamatórias sistêmicas, sugerindo que os EAA podem induzir aterosclerose por inflamação coronária e sistêmica.

**Figura Central:**
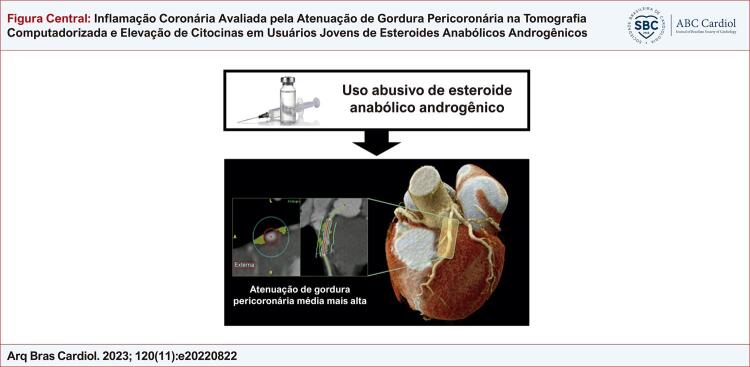
: Inflamação Coronária Avaliada pela Atenuação de Gordura Pericoronária na Tomografia Computadorizada e Elevação de Citocinas em Usuários Jovens de Esteroides Anabólicos Androgênicos

## Introdução

O uso abusivo de esteroides anabólicos androgênicos (EAA) tem sido associado a distúrbios no sistema cardiovascular. ^1 - 5^ Estudos prévios mostraram que o uso ilícito de EAA causa redução nos níveis plasmáticos de lipoproteína de alta densidade (HDL) e no efluxo de colesterol mediado pelo HDL, ^6^ e doença arterial coronariana (DAC) em usuários de EAA jovens, do sexo masculino. ^6 - 8^

A inflamação é a chave para o processo aterogênico associado à vulnerabilidade da placa aterosclerótica, que pode levar ao aumento do risco de eventos cardiovasculares, tais como síndrome coronária aguda e isquemia. ^9^ Em 2017, Antonopoulos et al. ^10^ desenvolveram uma métrica alternativa chamada Índice de Atenuação da Gordura perivascular (IAGp), que avalia inflamação coronária a partir do tecido adiposo vascular coronário. ^10^ Essa técnica permite a detecção fenotípica induzida pela inflamação vascular mesmo antes do início da doença aterosclerótica.

Gaibazzi et al. ^11^ mostraram que os pacientes com infarto do miocárdio e artérias coronárias não obstrutivas e valores do IAGp aumentados, apresentam, com maior frequência, placas nas artérias coronárias em comparação aos indivíduos sadios controle. ^11^ Em um estudo prévio, ^9^ observamos que 25% dos usuários de EAA apresentavam pelo menos duas artérias coronárias com placas. Assim, podemos especular que o uso abusivo de EAA poderia levar a uma maior atenuação de gordura perivascular. Ainda, citocinas pró-inflamatórias, tais como interleucinas 1 e 6 (IL-1 e IL-6) e Fator de Necrose Tumoral alfa (TNF-α), estão associadas com alteração fenotípica no tecido perivascular coronariano. ^10^ No entanto, a associação entre Atenuação de Gordura Pericoronária (AGP) e citocinas pró-inflamatórias em usuários de EAA não é conhecida.

A hipótese do presente estudo é que EAA causa DAC por inflamação sistêmica e da artéria coronária. Nosso objetivo foi investigar a associação da inflamação da artéria coronária, avaliada pela AGP média (AGPm), com inflamação sistêmica, avaliada pelos níveis de citocinas no sangue periférico, em usuários e em não usuários de EAA.

## Materiais e métodos

### População do estudo

O estudo foi aprovado pelo comitê de ética local para pesquisa envolvendo seres humanos (4958/19/177).

Este estudo é uma análise secundária, com delineamento transversal, de um estudo prévio. ^6^ Cinquenta participantes pareados por idade entre 18 e 45 anos de idade foram incluídos no estudo: 20 usuários de EAA (grupo UEAA) e 20 não usuários de EAA (grupo NUEAA). Ambos os grupos (grupos UEAA e NUEAA) eram levantadores de peso ou fisioculturistas, recrutados de academias. Além disso, 10 homens sedentários, pareados por idade (que não praticavam esportes e/ou exercício físico regularmente, ou atividade física como caminhada com intensidade leve/moderada <150 minutos por semana), sem doença cardiovascular – hipertensão, diabetes, hipercolesterolemia, obesidade [Índice de Massa Corporal (IMC) > 30 Kg/m ^2^ ] foram incluídos no grupo controle. Critérios de exclusão foram tabagismo, consumo de álcool, uso de diuréticos, estatinas e/ou anti-hipertensivos, doença hepática, e doença renal.

Os grupos UEAA e NUEAA haviam se envolvido em treinamento de força por pelo menos dois anos. Os UEAA deveriam autoadministrar EAA em ciclos periódicos com duração de oito a 12 semanas por pelo menos dois anos, com 2-4 ciclos por ano. Todos os usuários estavam em um ciclo durante o estudo.

### Medidas e procedimentos

Todos os participantes se abstiveram de suplementos esportivos, produtos contendo cafeína, e treino de exercício físico por 48 horas antes dos exames.

### Atenuação de gordura pericoronária

Previamente, todos os participantes foram submetidos a uma angiotomografia computadorizada de acordo com as diretrizes da Sociedade de Tomografia Computadorizada Cardiovascular ( *Society of Cardiovascular Computed Tomography, SCCT* ). ^12^ Todas as imagens de Tomografia Computadorizada (TC) foram adquiridas em um tomógrafo com 320 fileiras de detectores (Aquillion OneTM – Toshiba Medical Systems Corporation, Otawara, Japão) com cortes de 0,5 mm de espessura conforme descrito anteriormente. ^6^ Para a análise da atenuação de gordura perivascular média, utilizamos o programa TeraRecon (TeraRecon Aquarius 4.4.12.249 inc.), e usamos a fluxo pós-processamento da artéria coronária. Para melhor visualização, utilizamos a melhor fase diastólica, sem artefatos de movimento; eventualmente, necessitamos usar outras fases para uma melhor definição da parede do vaso. Com um pacote de fluxo de trabalho cardíaco, mudamos os parâmetros para acompanhar a visualização perivascular conforme sugerido na literatura; analisamos 5mm ao redor da parede do vaso (parede mais externa) e definimos 40mm de extensão da artéria descendente anterior (ADA) esquerda, artéria circunflexa (ACX) esquerda, e artéria coronária direita (ACD). Na ACD, nós excluímos 10mm proximal à aorta para evitar invasão da região perivascular da aorta. Para análise da AGPm, selecionamos os limiares -190 à -30HU. ^10 , 13 - 17^ As análises foram realizadas por dois observadores cegos (C.E.R., 20 anos de experiência, e D.C.S., três anos de experiência). Discrepâncias foram resolvidas por consenso entre os dois observadores experientes.

### Perfil inflamatório

As amostras de sangue foram coletadas pela manhã (entre 8h e 10h) após 12 horas de jejum e após 30 minutos de repouso. Kits de ensaio de imunoadsorção enzimática (ELISA) foram usados para medir IL-1, IL-6, IL-10 e TNF-α. As placas de ELISA foram sensibilizadas com os marcadores, em tampão carbonato, 50µL por poço, e incubado durante a noite a 4 ^o^ C. O marcador foi usado a uma concentração de 2μg/mL; 50 μL de anticorpo foi adicionado a cada poço a uma concentração de 0,5μg/mL, preparado em solução de lavagem, e incubado por uma hora a 37 ^o^ C. Os resultados foram obtidos com base na leitura da densidade ótica em um espectrofotômetro com um filtro de 450nm como descrito anteriormente. ^18^ As análises de interleucina foram realizadas em duplicata, e a média entre as medidas foi usada no estudo.

### Composição corporal

A composição corporal foi avaliada por Absorciometria de Fóton Duplo (DXA, Discovery DXA system, Hologic Inc) para medir massa livre de gordura, massa de gordura, e porcentagem de gordura em todos os participantes. Todas as medidas do DXA foram realizadas pelo mesmo técnico, e o erro de precisão foi estabelecido de acordo com os padrões do *International Society for Clinical Densitometry* . O DXA foi usado para excluir possível viés do IMC entre os participantes.

### Análise estatística

Os dados são apresentados em média ± Desvio Padrão (DP) ou mediana [Intervalo Interquartil (IIQ) 25-75%], de acordo com a normalidade dos dados. O teste Kolmogorov-Smirnov foi usado para avaliar a distribuição normal das variáveis estudadas. Os dados paramétricos foram comparados pelo teste ANOVA *one-way* . Quando uma diferença significativa foi encontrada, o teste comparativo de Scheffé foi usado. Os testes de comparação múltipla Kruskal Wallis e de Dunn foram usados para os dados não paramétricos. O teste de correlação bivariada (Spearman) também foi usado, e um p<0,05 foi usado para indicar significância estatística. O programa SPSS ( *Statistical Package for the Social Sciences* ), versão 23, foi usado para realizar as análises.

## Resultados

As características físicas e o perfil lipídico dos pacientes estão descritos na Tabela 1 . O grupo UEAA apresentou maior peso corporal, IMC, e massa magra em comparação aos grupos NUEAA e controle. Não foi encontrada diferença significativa de idade ou altura entre os grupos UEAA, NUEAA e controle. Além disso, o grupo UEAA apresentou concentrações plasmáticas de lipoproteína de alta densidade (HDL) mais baixas e de lipoproteína de baixa densidade (LDL) mais altas em comparação ao grupo NUEAA e grupo controle.

**Tabela 1:** – Características físicas e perfil lipídico de usuários de esteroides anabólicos androgênicos (UEAA), não usuários de esteroides anabólicos androgênicos (NUEAA), e controles sedentários (CS)

Variáveis	UEAA = 20	NUEAA = 20	CS = 10	p
Idade (anos)	29 ± 5	29 ± 5	29 ± 3	0,861
Altura (m)	1,78 ± 0,04	1,80 ± 0,09	1,76± 0,08	0,841
Peso (Kg)	97,4 (90,1-104,9) *†	82,0 (74,0-88,0)	74,8 (70,0-87,5)	<0,001
IMC (Kg/m ^2^ )	31,11 ± 3,45 *†	25,45 ± 1,92	25,70 ± 3,38	<0,001
Massa de gordura (%)	13,18 ± 5,62 *†	19,27 ± 4,33 *	27,59 ± 7,49	0,005
Massa magra (kg)	82,05 ± 9,18 *†	62,81 ± 7,15 *	53,94 ± 7,38	<0,001
CT (mg/dL)	186 (143-208)	155 (135-188)	189 (175-200)	0,070
HDL-c (mg/dL)	19 (13-25) *†	44 (41-54)	50 (40-55)	<0,001
LDL-c (mg/dL)	144 (105-179) *†	96 (81-125)	122 (105-132)	0,001
TG (mg/dL)	74 ± 23	75 ± 35	98 ± 45	0,151
Glicose (mg/dL)	90 ± 7	90 ± 6	92 ± 8	0,661

IMC: índice de massa corporal; CT: colesterol total; HDL-c: lipoproteína de alta densidade; LDL-c: lipoproteína de baixa densidade; TG: triglicerídeos; * p <0,05 vs. CS; † p< 0.05 vs. NUEAA.

**Figura 1:**
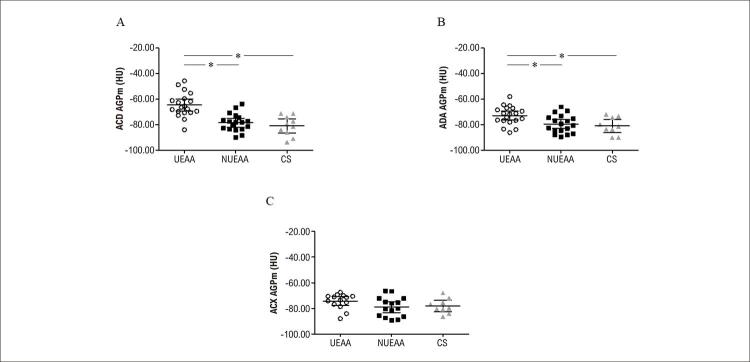
– A) Atenuação de gordura pericoronária média (AGPm) na artéria coronária direita (ACD); B) Artéria descendente anterior (ADA) esquerda; C) e artéria circunflexa (ACX) em usuários de esteroides anabólicos androgênicos (UEAA), Não usuários de esteroides anabólicos androgênicos (NUEAA), e controles sedentários (CS) * = p<0,05.

O grupo UEAA apresentou maior AGPm na ACD em comparação aos grupos NUEAA e controle [-65,87 (70,51-60,70) vs. -78,07 (83,66-72,87) vs. -78,46 (85,41-71,99) HU, respectivamente, p<0,05] ( Figura 1A ). Além disso, a AGPm na ADA foi mais alta em comparação a NUEAA e ao grupo controle [-71,47 (76,40-66,61) vs. -79,32 (84,37-74,59) vs. -82,52 (88,44-75,81) HU, respectivamente, p<0,05] ( Figura 1B ). Contudo, não observamos diferença na AGPm na ACX entre os grupos UEAA, NUEAA, e controle [-72,41 (77-70) vs. -80,13 (86,22-72,23) vs. -78,29 (80,63-72,29) HU, respectivamente, p>0,05] ( Figura 1C ). Análise da gordura pericoronária proximal da ADA pode ser vista na Figura Central .

**Figura 2:**
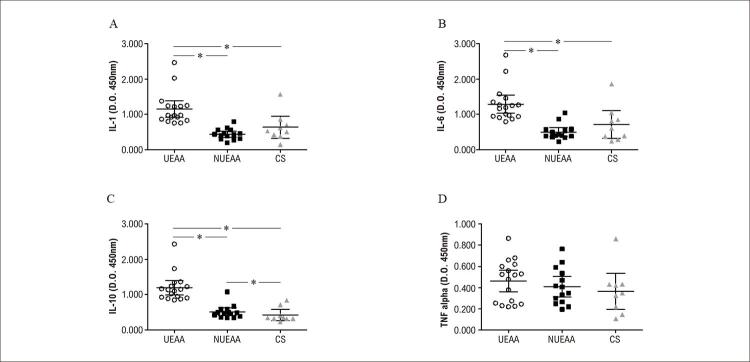
– A) Concentração de interleucina (IL)-1 (IL-1); B) IL-6, C) IL-10, e D) fator de necrose tumoral alfa (TNF-α) usuários de esteroides anabólicos androgênicos (UEAA), não usuários de esteroides anabólicos androgênicos (NUEAA), e controles sedentários (CS) * = p<0,05.

**Figura 3:**
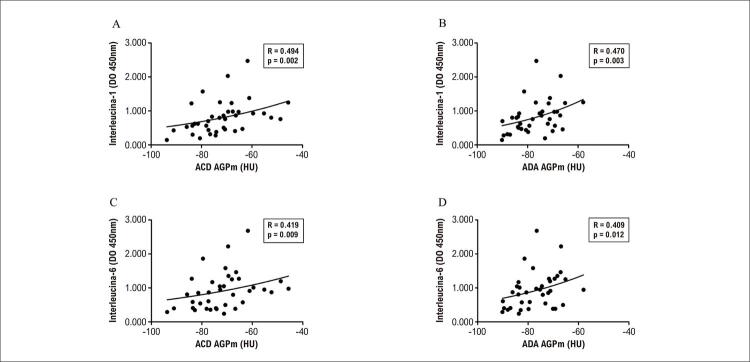
– A) Correlação positiva entre Interleucina-1 (IL-1) e atenuação de gordura pericoronária média (AGPm) na artéria coronária direita (ACD); B) IL-1 e AGPm na artéria descendente anterior (ADA) esquerda; C) IL-6 e AGPm na ACD; e D) AGPm na ADA esquerda; DO: densidade ótica.

Níveis de IL-1, IL-6, e IL-10 foram significativamente maiores no grupo UEAA em comparação aos níveis nos grupos NUEAA e controle (Figura 2A-C, respectivamente). No entanto, os níveis de TNF-α não foram diferentes entre os três grupos ( Figura 2D ). Outras análises mostraram uma correlação positiva entre IL-1 e AGPm na ACD; IL-1 e AGPm na ADA; IL-6 e AGPm na ACD; e IL-6 e AGPm na ADA ( Figuras 3A-D , respectivamente).

Além disso, o tempo médio de uso de EAA no grupo UEAA foi 8 ± 6 anos. No grupo NUAEE, 20%, 40%, 35%, e 5% deles usavam dois três, quatro, e cinco diferentes tipos de AEE, respectivamente. Os tipos mais comuns de EAA usados foram testosterona (enantato, propionato, undecilato, e cipionato), nandrolona, boldenona, trembolona, e estanozolol. A razão testosterona/epitestosterona foi mais alta no grupo UEAA que nos grupos NUEAA e controle (63,8±44,6 vs. 0,9±1,2 vs. 0,9±0,9, respectivamente, *p* <0,05). Os grupos UEAA e NUEAA se envolveram em treinamento de força por aproximadamente 10 anos.

## Discussão

Neste estudo caso-controle comparando a AGPm e citoquinas periféricas nos grupos UEAA, NUEAA e controle, encontramos: i. AGPm elevada no grupo UEAA; citocinas elevadas no grupo UEAA, e iii. AGPm foi associada com citocinas periféricas no grupo UEAA.

A inflamação vascular é o gatilho para o desenvolvimento de placa aterosclerótica coronária e é uma característica típica de ruptura da placa aterosclerótica. ^9^ Antonopoulos et al. ^10^ relataram que a artéria coronária inflamada se difunde ao tecido adiposo perivascular, que altera a composição da gordura perivascular ao redor das artérias inflamadas. Essas alterações levam à atenuação ao redor das artérias coronárias da atenuação lipídica típica – valores de HU mais negativos (por exemplo, mais próximo a -190 HU) – à fase mais aquosa – valores de HU menos negativos (por exemplo, mais próximo a -30 HU). ^10^ Embora sejam necessários mais estudos para confirmar os valores de HU como um marcador de inflamação coronária, um estudo prévio sugeriu que um valor de -70HU parece ser um ponto de corte razoavelmente robusto para definir inflamação das artérias coronárias. ^11^ Ainda, Oikonomou et al. ^19^ sugeriram que valores de IAGp (ponto de corte - 70 HU) são um indicador de mortalidade cardíaca aumentada. ^19^

Gaibazzi et al. ^11^ mostraram que pacientes com infarto do miocárdio, na ausência de estenose coronária obstrutiva e valores de IAGp menores que -70HU (aproximadamente -68,37 HU), apresentam placas na artéria coronária com maior frequência em comparação a controles sadios que apresentam IAGp maior que -70 (aproximadamente -78,03HU). Em nosso estudo, encontramos que UEAA apresentaram uma AGPm (ACD e ADA) de aproximadamente -68,71HU. Esse dado está de acordo com nossas observações prévias de que cerca de um a quatro fisiculturistas (25%) que usaram EAA apresentaram sinais de DAC subclínica na angiotomografia computadorizada. Em contraste, nenhum dos NUEAA ou dos participantes sedentários apresentaram DAC subclínica. ^6^

A aterosclerose é uma doença inflamatória crônica. ^9^ Está bem estabelecido que os marcadores de inflamação sistêmica, tais como IL-1, IL-6, e TNF-α, são os principais fatores que atuam ao longo da cascata inflamatória vascular. ^20 , 21^ Em nosso estudo, encontramos concentrações mais altas de IL-1 e IL-6 no grupo UEAA em comparação aos grupos NUEAA e controle. Apesar do aumento das citocinas pró-inflamatórias, o IL-10 foi mais alto em UEAA que em NUEAA e controles. A IL-10 é a principal citocina anti-inflamatória com um importante papel na modulação e na produção de TNF-α. ^22^ Nossos resultados sugerem que a IL-10 pode equilibrar o perfil pró-inflamatório/anti-inflamatório. Essa hipótese deve ser abordada em estudos futuros.

O uso abusivo de EAA tem sido associado com diminuição no efluxo de colesterol mediado pelo HDL e nos níveis plasmáticos de HDL, e aumento nas concentrações de LDL plasmática. Esse metabolismo lipídico alterado é um dos mecanismos envolvidos no processo aterogênico. Ainda, uma alteração na taxa de cisalhamento, caracterizada por uma taxa retrógrada e oscilatória, está associada com inflamação, aterosclerose, e disfunção endotelial. ^23^ A proteína C reativa ultrassensível (PCR-us) também é um marcador de inflamação vascular. ^24^ Nós relatamos em um estudo prévio ^25^ que UEAA apresentam uma taxa de cisalhamento retrógrada e oscilatória na artéria braquial e níveis mais altos de PCR-us em comparação a NUEAA. ^25^ Todas essas alterações podem estar associadas à inflamação coronária e ao desenvolvimento prematuro de doença aterosclerótica em UEAA jovens.

### Implicação clínica

A implicação clínica de nossos resultados é que a atenuação de gordura pericoronária, medida por angiotomografia coronária pode ser usada para avaliar a carga de inflamação coronária e, assim, avaliar o risco cardiovascular de uma população específica. A inflamação coronária pode ser identificada por AGPm mesmo antes que placas ateroscleróticas possam ser detectadas. Assim, a AGPm é um potencial biomarcador precoce de DAC em UEAA. Serão necessários estudos maiores, longitudinais, para avaliar a aplicação clínica rotineira dessa nova tecnologia.

### Limitações

Reconhecemos as limitações em nosso estudo. Estudamos uma amostra pequena, e os resultados são uma subanálise de um estudo anterior. ^6^ Utilizamos a AGPm pelo TeraRecon conforme descrito por Nomura et al. ^26^ e os valores não podem ser comparados diretamente com outras abordagens à inflamação pericoronária. Os mecanismos envolvidos na atenuação de gordura pericoronária estavam foram do escopo de nosso estudo. A correlação observada em nosso estudo deve ser interpretada com cuidado, e o mecanismo de atenuação de gordura pericoronária deve ser abordada em estudos futuros.

## Conclusão

Este estudo indica que o uso abusivo de EAA está associado com inflamação coronária e níveis mais altos de citocinas inflamatórias sistêmicas. Ainda, a atenuação de gordura pericoronária pode ser usada para rastrear inflamação coronária (como um biomarcador de DAC) mesmo antes do desenvolvimento de placas visíveis.
