# Supplementary material for: Inflamação Coronária Avaliada pela Atenuação de Gordura Pericoronária na Tomografia Computadorizada e Elevação de Citocinas em Usuários Jovens de Esteroides Anabólicos Androgênicos
Source: Arq Bras Cardiol. 2023 Nov 13;120(11):e20220822. [Article in Portuguese] doi: 10.36660/abc.20220822 (PMC10697680; doi:10.36660/abc.20220822)
Supplement: Translation

# Translation: Coronary Inflammation by Computed Tomography Pericoronary Fat Attenuation and Increased Cytokines in Young Male Anabolic Androgenic Steroid Users

**Authors:** Francis Ribeiro de Souza, Carlos E. Rochitte, Douglas Carli Silva, Barbara Sampaio, Marisa Passarelli, Marcelo R. dos Santos, Guilherme W. Fonseca, Antonio Carlos Battaglia-Filho, Kelly Correa, Renata Margarida do Val, Maurício Yonamine, Rosa Maria R. Pereira, Carlos Eduardo Negrão, Roberto Kalil-Filho, Maria Janieire de Nazaré Nunes Alves

**Affiliations:** 1 Brazil Instituto do Coração do Hospital das Clínicas da Faculdade de Medicina da Universidade de São Paulo, São Paulo, SP – Brazil; 2 Brazil Faculdade de Medicina da Universidade de São Paulo (FMUSP), São Paulo, SP – Brazil; 3 Brazil Instituto de Medicina Tropical de São Paulo, São Paulo, SP – Brazil; 4 Brazil Universidade de São Paulo – Faculdade de Medicina – Laboratório de Lípides, São Paulo, SP – Brazil; 5 Brazil Universidade Nove de Julho, São Paulo, SP – Brazil; 6 Brazil Hospital Israelita Albert Einstein, São Paulo, SP – Brazil; 7 Brazil Universidade de São Paulo – Faculdade de Ciências Farmacêuticas, São Paulo, SP – Brazil; 8 Brazil Hospital Sírio Libanês, São Paulo, SP – Brazil

**Keywords:** Coronary Artery Disease, Pericoronary Fat Attenuation, Anabolic Androgenic Steroids

## Abstract

**Background:**

Anabolic androgenic steroid (AAS) abuse has been associated with coronary artery disease (CAD). Pericoronary fat attenuation (pFA) is a marker of coronary inflammation, which is key in the atherosclerotic process.

**Objective:**

To evaluate pFA and inflammatory profile in AAS users.

**Methods:**

Twenty strength-trained AAS users (AASU), 20 AAS nonusers (AASNU), and 10 sedentary controls (SC) were evaluated. Coronary inflammation was evaluated by mean pericoronary fat attenuation (mPFA) in the right coronary artery (RCA), left anterior descending coronary artery (LAD), and left circumflex (LCx). Interleukin (IL)-1 (IL-1), IL-6, IL-10, and TNF-alpha were evaluated by optical density (OD) in a spectrophotometer with a 450 nm filter. P<0.05 indicated statistical significance.

**Results:**

AASU had higher mPFA in the RCA (-65.87 [70.51-60.70] vs. -78.07 [83.66-72.87] vs.-78.46 [85.41-71.99] Hounsfield Units (HU), respectively, p<0.001) and mPFA in the LAD (-71.47 [76.40-66.61] vs. -79.32 [84.37-74.59] vs. -82.52 [88.44-75.81] HU, respectively, p=0.006) compared with AASNU and SC. mPFA in the LCx was not different between AASU, AASNU, and SC (-72.41 [77.17-70.37] vs. -80.13 [86.22-72.23] vs. -78.29 [80.63-72.29] HU, respectively, p=0.163). AASU compared with AASNU and SC, had higher IL-1, (0.975 [0.847-1.250] vs. 0.437 [0.311-0.565] vs. 0.530 [0.402-0.780] OD, respectively, p=0.002), IL-6 (1.195 [0.947-1.405] vs. 0.427 [0.377-0.577] vs. 0.605 [0.332-0.950] OD, p=0.005) and IL-10 (1.145 [0.920-1.292] vs. 0.477 [0.382-0.591] vs. 0.340 [0.316-0.560] OD, p<0.001). TNF-α was not different between the AASU, AASNU, and SC groups (0.520 [0.250-0.610] vs. 0.377 [0.261-0.548] vs. 0.350 [0.182-430]), respectively.

**Conclusion:**

Compared with ASSNU and controls, AASU have higher mPFA and higher systemic inflammatory cytokines profile suggesting that AAS may induce coronary atherosclerosis through coronary and systemic inflammation.

**Central Illustration:** : Coronary Inflammation by Computed Tomography Pericoronary Fat Attenuation and Increased Cytokines in Young Male Anabolic Androgenic Steroid Users

## Introduction

Anabolic androgenic steroid (AAS) abuse has been associated with disturbances in the cardiovascular system.^1 - 5^Previous studies have shown that illicit use of AAS leads to decreased high-density lipoprotein (HDL) plasma concentration and cholesterol efflux mediated by HDL,^6^and coronary artery disease (CAD) in young male AAS users.^6 - 8^

Inflammation is the key to the atherogenic process associated with atherosclerotic plaque vulnerability, which can lead to higher risk of cardiovascular events, such as acute coronary syndrome and ischemia.^9^In 2017, Antonopoulos et al.^10^developed an alternative metric called perivascular fat attenuation index (pFAI), that evaluate coronary inflammation by coronary perivascular adipose tissue.^10^This technique allows the phenotypic detection induced by vascular inflammation even before the onset of atherosclerotic disease.

Gaibazzi et al.^11^showed that patients with myocardial infarction with non-obstructive coronary arteries and increased pFAI values, more frequently have coronary artery plaques compared with healthy controls.^11^Previously,^9^we reported that 25% of AAS users had at least two coronary arteries with plaques. Thus, it is possible to speculate that AAS abuse could lead to higher perivascular fat attenuation. Furthermore, pro-inflammatory cytokines, such as interleukins 1 and 6 (IL-1 and IL-6) and tumour necrosis factor alpha (TNF-α), are associated with phenotypic alteration in the coronary perivascular tissue.^10^However, the association between pericoronary fat attenuation (PFA) and pro-inflammatory cytokines in AAS users is unknown.

The present study hypothesized that AAS leads to CAD through coronary and systemic inflammation. We aimed to investigate the association of coronary inflammation, assessed by mean PFA (mPFA), with systemic inflammation, assessed by peripheral blood cytokines in AAS users and non-users.

## Materials and methods

### Study population

The local committee for the Protection of Human Subjects approved this study (4958/19/177).

This is a cross-sectional, secondary analysis from a previous study.^6^Fifty age-matched participants between 18 and 45 years were included in the study: 20 AAS users (group AASU) and 20 AAS nonusers (group AASNU). Both groups (AASU and AASNU) were recreational weightlifters or amateur bodybuilding athletes who were recruited from gyms. In addition, 10 age-matched sedentary men (without regular exercise training and/or sports, <150 min/week of physical activity such as walking with light/moderate intensity) without cardiovascular disease – hypertension, diabetes, hypercholesterolemia, obesity (body mass index [BMI] >30 Kg/m^2^) were included as the control group. Exclusion criteria were smoking, alcohol consumption, use of diuretics, statins and/or anti- hypertensive medications, liver disease, and kidney disease.

AASU and AASNU groups had been involved in strength training for at least two years. AASU should be self-administering AAS in periodic cycles lasting from eight to 12 weeks for at least two years with 2-4 cycles per year. All AASU were on a cycle over the course of the study.

### Measures and procedures

All the participants refrained from sports supplements, caffeine-containing products, and exercise training for 48 hours before the exams.

### Pericoronary fat attenuation

Previously, all participants underwent coronary computed tomography angiography according to the guidelines of the Society of Cardiovascular Computed Tomography (SCCT).^12^All computed tomography (CT) scans were acquired in a 320-row detector scanner (Aquillion One^TM^– Toshiba Medical Systems Corporation, Otawara, Japan) with 0.5-mm thick slices as described previously.^6^For the mPFA analysis, we used the dedicated software TeraRecon (TeraRecon Aquarius 4.4.12.249 inc.) and applied it to the coronary post-processing workflow. For a better view, we used the best diastolic phase with no motion artifacts; eventually we needed to use other phases to improve vessel wall definition. With a cardiac workflow package and plaque analysis, we changed the parameters to follow the reference perivascular visualization as suggested by the literature; we looked 5mm around the vessel wall (outer vessel wall) and defined the 40mm proximal vessel extension of left anterior descending coronary artery (LAD), left circumflex artery (LCx), and right coronary artery (RCA). On the RCA, we excluded 10mm proximal to the aorta to avoid encroachment on the perivascular aorta region. To check mPFA, we selected the -190 to -30 HU threshold.^10 , 13 - 17^The analyses were performed by two blinded observers (C.E.R., 20 years of experience and D.C.S., three years of experience). Discrepancies were solved by consensus between the two experienced observers.

### Inflammatory profile

Blood samples were collected in the morning (between 8:00-10:00 A.M.) after 12h fasting and after 30 minutes of resting. High-sensitive enzyme-linked immunosorbent assay (ELISA) kits were used to measure IL-1, IL-6, IL-10, and TNF-α. The ELISA plates were sensitized with the markers, in carbonate buffer, 50 μL per well, and incubated overnight at 4º Celsius. The marker was used at a concentration of 2μg/mL; 50 μL of detector antibody was added to each well to a concentration of 0.5μg/mL, prepared in washing liquid, and incubated for one hour at 37º Celsius. The results were obtained based on the reading of the optical density in a spectrophotometer with a 450 nm filter as described previously.^18^The interleukin analyses were performed in duplicate, and the mean was used in this study.

### Body composition

Body composition was assessed by dual-energy X-ray absorptiometry (DXA), (Discovery DXA system, Hologic Inc) to measure total fat-free mass, fat mass, and fat percentage in all participants. All DXA measurements were performed by the same experienced technician and the precision error was established according to the International Society for Clinical Densitometry’s standards. DXA was used to exclude possible bias of BMI among the participants.

### Statistical analysis

Data are presented as mean ± standard deviation (SD) or median (interquartile range [IQR] 25-75%) according to data normality. The Kolmogorov-Smirnov test was used to evaluate the normal distribution of the variables studied. The parametric data were compared through one-way ANOVA. When a significant difference was found, the Scheffé post-hoc comparison test was used. Kruskal Wallis and Dunn’s multiple comparison tests were used for nonparametric data. Bivariate correlation test (Spearman) was also used. A P<0.05 indicated statistical significance. The Statistical Package for the Social Sciences (SPSS) version 23 was used to perform all the statistical analyses.

## Results

Physical characteristics and lipid profile are described in Table 1 . AASU had higher weight, BMI, and lean mass compared with AASNU and controls. Body fat was lower in AASU compared with that in AASNU and controls. No significant difference was found in age and height between AASU, AASNU, and control groups. In addition, the AASU had lower high-density lipoprotein cholesterol (HDL-c) plasma levels and higher low density lipoprotein cholesterol (LDL-c) plasma levels compared with AASNU and controls.

**Table 1:** – Physical characteristics and lipid profile in anabolic androgenic steroid users (AASU), anabolic androgenic steroid nonusers (AASNU), and sedentary control (SC)

Variables	AASU = 20	AASNU = 20	SC = 10	p
Age (years)	29 ± 5	29 ± 5	29 ± 3	0.861
Height (m)	1.78 ± 0.04	1.80 ± 0.09	1.76± 0.08	0.841
Weight (kg)	97.4 (90.1-104.9) *†	82.0 (74.0-88.0)	74.8 (70.0-87.5)	<0.001
BMI (kg/m^2^)	31.11 ± 3.45 *†	25.45 ± 1.92	25.70 ± 3.38	<0.001
Fat mass (%)	13.18 ± 5.62 *†	19.27 ± 4.33 *	27.59 ± 7.49	0.005
Lean mass (kg)	82.05 ± 9.18 *†	62.81 ± 7.15 *	53.94 ± 7.38	<0.001
TC (mg/dL)	186 (143-208)	155 (135-188)	189 (175-200)	0.070
HDL-c (mg/dL)	19 (13-25) *†	44 (41-54)	50 (40-55)	<0.001
LDL-c (mg/dL)	144 (105-179) *†	96 (81-125)	122 (105-132)	0.001
TG (mg/dL)	74 ± 23	75 ± 35	98 ± 45	0.151
Glucose (mg/dL)	90 ± 7	90 ± 6	92 ± 8	0.661

AASU: anabolic androgenic steroids users; AASNU: anabolic androgenic steroids nonusers; SC: sedentary control; BMI: body mass index; TC: total cholesterol; HDL-c: high-density lipoprotein cholesterol; LDL-c: low-density lipoprotein cholesterol. TG: triglycerides * p <0.05 vs. SC; † p< 0.05 vs. AASNU.

AASU had higher mPFA in RCA compared with AASNU and controls [-65.87 (70.51-60.70) vs. -78.07 (83.66-72.87) vs.-78.46 (85.41-71.99) HU, respectively, p<0.05] ( Figure 1A ). Additionally, the mPFA in the LAD was higher in AASU compared with AASNU and controls [-71.47 (76.40-66.61) vs. -79.32 (84.37-74.59) vs. -82.52 (88.44-75.81) HU, respectively, p<0.05] ( Figure 1B ). However, no difference was found in mPFA in the LCx between AASU, AASNU, and controls [-72.41 (77-70) vs. -80.13 (86.22-72.23) vs. -78.29 (80.63-72.29) HU, respectively, p>0.05] ( Figure 1C ). Analysis of proximal perivascular fat atternuation in the LAD can be seen in Central Illustration .

**Figure 1:** – A) Mean pericoronary fat attenuation (mPFA) in the right coronary artery (RCA); B) left anterior descending coronary artery (LAD); C) and left circumflex artery (LCx) in anabolic androgenic steroid users (AASU), anabolic androgenic steroid nonusers (AASNU), and sedentary control (SC). * = p<0.05.

IL-1, IL-6, and IL-10 were significantly higher in AASU compared with that in AASNU and SC ( Figure 2A-C , respectively). However, TNF-α was not different between the three groups ( Figure 2D ). Further analysis showed a positive correlation between IL-1 and RCA pFAM; IL-1 and LAD pFAM; IL-6 and RCA pFAM; IL-6 and LAD mPFA ( Figures 3A-D , respectively).

**Figure 2:** – A) Concentration of interleukin (IL)-1 (IL-1); B) IL-6, C) IL-10, and D) tumor necrosis factors alpha (TNF-α) in anabolic androgenic steroid users (AASU), anabolic androgenic steroid nonusers (AASNU), and sedentary control (SC); OD: optical density * = p<0.05.

**Figure 3:** – A) Positive correlation between interleucin-1 (IL-1) and right coronary artery (RCA) mean pericoronary fat attenuation (mPFA); B) IL-1 and mPFA in the left anterior descending coronary artery (LAD); C) IL-6 and mPFA in the RCA; IL-6, and D) mPFA in the LAD; OD: optical density.

In addition, the average time of AAS use in the AASU group was 8±6 years. Within the AASU group, 20%, 40%, 35%, and 5% of them were using two, three, four, and five different types of AAS, respectively. Testosterone (enanthate, propionate, undecylate, and cypionate), nandrolone, boldenone, trenbolone, and stanozolol were the most common types of AAS used in AASU. The testosterone/epitestosterone (T/E) ratio was higher in AASU compared with that in AASNU and SC (63.8±44.6 vs. 0.9±1.2 vs. 0.9±0.9, respectively, *p* <0.05). The AASU and AASNU groups had been involved in strength training for approximately 10 years.

## Discussion

In this case-control study comparing mPFA and peripheral cytokines in AASU, AASNU and controls, we found: i. High mPFA in AASU; ii. High cytokines in AASU, and iii. mPFA was associated with peripheral cytokines.

Vascular inflammation is the trigger for coronary atherosclerotic plaque development and is a typical feature of atherosclerotic plaque rupture.^9^Antonopoulos et al.^10^reported that the inflamed coronary artery diffuses to the perivascular adipose tissue, which changes the composition of perivascular fat around inflamed arteries. These changes lead to attenuation around coronary arteries from typical lipid attenuation (more negative HU values [e.g., closer to –190 HU]) to the more aqueous phase (less negative HU values [e.g., closer to –30 HU]).^10^Although more studies are needed to confirm the HU values as a marker of coronary inflammation, a previous study^11^suggested that -70 HU appears to be a reasonably robust cutoff to define the inflammation of coronary arteries. Moreover, Oikonomou et al.^19^suggested that high perivascular FAI values (cutoff ≥ -70.1 HU) are an indicator of increased cardiac mortality.^19^

Gaibazzi et al.^11^showed that patients with myocardial infarction in the absence of obstructive coronary stenosis and pFAI values lower than -70 HU values (approximately -68.37 HU) more frequently have coronary artery plaques compared with healthy controls who have pFAI higher than -70 (approximately -78.03 HU).^11^In our study, we found that AASU had a mPFA (RCA and LAD) of approximately -68.71 HU. Interestingly, this finding is in line with our previous observations that about 1 in 4 weightlifters (25%) who used AAS had signs of subclinical CAD in the computed tomography angiography. In contrast, none of the AASNU or the sedentary participants had subclinical CAD.^6^

Atherosclerosis is a chronic inflammatory disease.^9^It is well established that markers of systemic inflammation, such as IL-1, IL-6, and TNF-α, are the major players in the downstream vascular inflammatory cascade.^20 , 21^In our study, we found a higher IL-1 and IL-6 concentration in AASU compared with AASNU and sedentary controls. However, no difference in TNF-α was found among groups. Despite the increased pro-inflammatory cytokines, IL-10 was higher in AASU compared with AASNU and sedentary controls.IL-10 is the main anti-inflammatory cytokine that plays an important role in modulating the production of TNF-α.^22^Our results suggest that IL-10 may balance the pro/anti-inflammatory profile. This hypothesis should be addressed in future studies.

AAS abuse has been associated with reduced cholesterol efflux mediated by HDL and HDL plasma concentration, and increased LDL plasma concentration. This altered lipid metabolism is one of the mechanisms involved in the atherogenic process. Furthermore, disturbed shear rate (SR), characterized by increased retrograde and oscillatory SR, is associated with inflammation, atherosclerosis, and endothelial dysfunction.^23^High-sensitivity C-reactive protein (hs-CRP) is also a marker of vascular inflammation.^24^We have previously^25^reported that AASU have increased retrograde and oscillatory SR in the brachial artery and higher hs-CRP compared with AASNU.^25^Taken together, all these alterations may be associated with coronary inflammation and premature development of atherosclerotic disease in young AASU.

### Clinical implication

The clinical implication of our results is that pericoronary fat attenuation measured by coronary computed tomography angiography can be used to assess the coronary inflammation burden and therefore assess the cardiovascular risk of a specific population. Coronary inflammation by mPFA can be detected even before detectable atherosclerotic plaque formation and, therefore, has the potential to become an early biomarker of CAD in AASU. The routine clinical application of this new technology will warrant further and larger longitudinal studies.

### Limitations

We recognize limitations in our study. We studied a small sample size, and the results are a subanalysis from a previous study.^6^We used mPFA by TeraRecon as previously described by Nomura et al.^26^and the values cannot be compared directly to other approaches to pericoronary inflammation. The mechanisms involved in pericoronary fat attenuation were out of the scope of our study. The correlation observed in our study should be interpreted with caution, and the mechanism of pericoronary fat attenuation should be addressed in future studies.

## Conclusion

This study indicates that AAS abuse is linked to coronary inflammation and higher systemic inflammatory cytokines profile. In addition, the pericoronary fat attenuation can be used to track coronary inflammation (as a biomarker of CAD) even before visible plaques develop.
